# Ternary Organic Solar Cells by Small Amount of Efficient Light Absorption Polymer PSEHTT as Third Component Materials

**DOI:** 10.3390/molecules28196832

**Published:** 2023-09-27

**Authors:** Han Zhang, Songrui Jia, Zhiyong Liu, Zheng Chen

**Affiliations:** 1 Institute of Physics and Electronic Information, Yunnan Normal University, Kunming 650500, China; 2 National and Local Joint Engineering Laboratory for Synthetic Technology of High Performance Polymer, College of Chemistry, Jilin University, Changchun 130012, China; jiasr21@mails.jlu.edu.cn; 3 Engineering Research Center of Special Engineering Plastics, Ministry of Education, College of Chemistry, Jilin University, Changchun 130012, China; 4 Key Laboratory of High Performance Plastics, Ministry of Education, College of Chemistry, Jilin University, Changchun 130012, China; 5 Yunnan Key Laboratory of Optoelectronic Information Technology, Kunming 650500, China; 6 Key Laboratory of Advanced Technique & Preparation for Renewable Energy Materials, Ministry of Education, Yunnan Normal University, Kunming 650500, China

**Keywords:** ternary OSCs, light absorption, exciton dissociation, carrier recombination

## Abstract

We prepared ternary organic solar cells (OSCs) by incorporating the medium wavelength absorption polymer PSEHTT into the PM6:L8-BO binary system. The power conversion efficiency (PCE) is improved from 15.83% to 16.66%. Although the fill factor (FF) is slightly reduced, the short-circuit current density (*J*_SC_) and open-circuit voltage (*V*_OC_) are significantly increased at the same time. A small amount of PSEHTT has a broad absorption spectrum in the short wavelength region and has good compatibility with PM6, which is conducive to fine-tuning the photon collection and improving the *J*_SC_. In addition, the highest occupied molecular orbital (HOMO) energy level of PSEHTT is deeper than that of PM6, which broadens the optical bandgap. This study provides an effective method to fabricate high-performance ternary OSCs by using a lower concentration of PSEHTT with PM6 as a hybrid donor material, which ensures a better surface and bulk morphology, improves photon collection, and broadens the optical bandgap.

## 1. Introduction

Solution-processed bulk-heterojunction (BHJ) organic solar cells (OSCs) have become indispensable in renewable energy due to the advantages of lightweight and flexibility as well as the potential for a large area roll-to-roll production [1,2,3]. Over the past two decades, the development of small molecule fullerene-free acceptors and medium band gap polymer donors has led to dramatic improvements in the power conversion efficiency (PCE) of photovoltaic devices [1,4]. Many well-known donor materials, such as “PM-series” polymer donors, can be combined with small-molecule fullerene-free acceptors. In recent years, narrow-bandgap non-fullerene acceptors extended the absorption spectra to the near-infrared region and upshifted or downshifted the energy levels compared with fullerene acceptors [5,6,7]. In addition, the “Y-series” small molecular fullerene free acceptor was first reported by Zou et al. and has led to many interesting results in organic photovoltaic [8]. Ding et al. synthesized a D18 polymer donor and achieved a PCE of over 18% [9]. Hou et al. reported a record PCE of approaches 19% with PBDB-T as the donor and blended HDC-4Cl and eC9 as acceptors [10].

It is well known that the common polymer donor and small molecules are non-fullerene acceptors with relatively narrow absorption windows and limit the photon harvesting of binary photoactive layers. Therefore, the absorption spectrum of binary photoactive (a blend of one donor and one acceptor) can hardly cover the whole solar spectrum (especially the short-wavelength light absorption), which is not conducive to efficient solar absorption and limits the further enhancement of photocurrent. Ternary OSCs (with a second donor or second acceptor as the third component material) can be easily fabricated and improve photon harvesting and exciton dissociation due to the different optical band gaps and good compatibility [11,12]. Second, acceptor-based ternary OSCs are often reported due to the different optical band gaps and good compatibility at the hybrid acceptor, while second donor-based ternary OSCs report less than second acceptor-based ternary OSCs due to the limited number of efficient donor materials and the difficulty better compatibility at the hybrid donor and acceptor [13,14].

While incorporating third component materials into binary films, it is inevitable to introduce charge traps at the D/A interface due to the lowest unoccupied molecular orbital (LUMO) energy level barriers of the two acceptors or highest occupied molecular orbital (HOMO) energy level barriers of the two donors [15,16]. The cascade LUMO energy levels or HOMO energy levels of the relevant materials should be taken into consideration to avoid forming deep charge traps [17,18]. The third component materials have complementary absorption spectra and improved exciton dissociation by adjusting the phase separation and surface morphology [19,20].

The PM6 and L8-BO show efficient medium wavelength and near-infrared wavelength light absorption, respectively. However, the PM6:L8-BO binary films have lost a large number of short-wavelength photons. Therefore, on the basis of not damaging the surface and bulk morphology of the photoactive layer film, further blue shifting the absorption peak and widening the band gap of the photosensitive layer films are also conducive to further increasing *J*_SC_ and *V*_OC_. In this manuscript, the efficient short wavelength light absorption polymer PSEHTT was used as the third component material, and we have designed a ternary OSC with a small amount of polymer donor (PSEHTT) as the third component material (guest donor), which possesses highly efficient light absorption and can generate cascade energy levels with the host donor (PM6) and acceptor (L8-BO). More efficient light absorption and exciton dissociation are achieved without destroying the crystallinity and surface and bulk morphology of the binary photoactive film (PM6:L8-BO). In all binary and ternary OSCs, the PSEHTT content in the hybrid donor (PM6:PSEHTT) was adjusted from 0 wt% to 15 wt%, and the weight ratio of the hybrid donor to the acceptor remained unchanged (1:1.4). The binary OSCs based on PM6:L8-BO had a PCE of 15.83% with a *V*_OC_ of 0.870 V, a *J*_SC_ of 24.39 mA cm^−2^, and an FF of 74.6%. Upon incorporation of PSEHTT into the PM6:L8-BO binary film, the ternary OSC showed a higher PCE of 16.66% (0.875 V for *V*_OC_, 25.66 mA cm^−2^ for *J*_SC_, and 74.2% for FF) compared to the PM6:L8-BO based binary OSC. The results demonstrate that constructing a ternary system by introducing a PSEHTT into the PM6:L8-BO binary film is an effective way to further improve the PCE of OSC.

## 2. Results and Discussions

**Figure 1:**
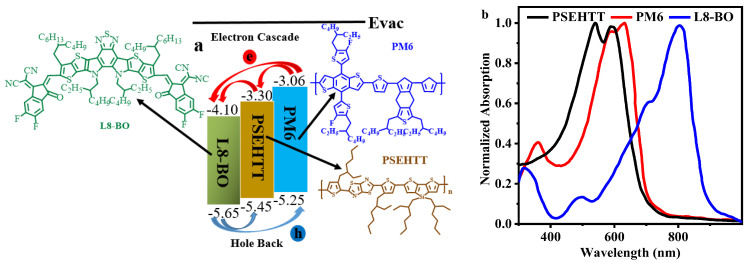
The chemical structure and energy levels (as shown in (**a**)) and normalized absorption (as shown in (**b**)) of the relevant materials [21,22,23].

Figure 1a shows the chemical structure and energy levels of the used materials. The chemical structure and energy levels of used materials are taken from the other paper.

The normalized UV-Vis absorption spectra of thin films of the neat materials are shown in Figure 1b. PSEHTT and PM6 show strong absorption at medium wavelengths. The absorption peak of PSEHTT is a significantly blue shift in favor of shorter wavelengths compared to PM6, which is beneficial to efficient absorption of short wavelength light.

**Figure 2:**
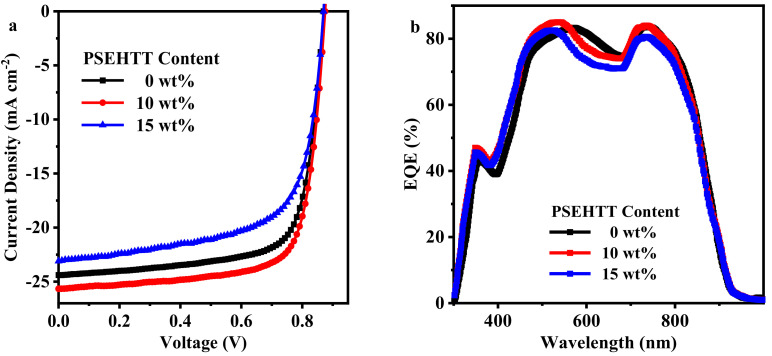
(**a**) The *J*-*V* curve and (**b**) EQE curve of three typical OSCs.

The current density-voltage (*J*-*V*) curves and external quantum efficiency (EQE) spectra of three typical OSCs [PM6:L8-BO binary OSCs, optimized ternary OSCs (ratio of PM6:PSEHTT is 0.9:0.1) and high PSEHTT ternary OSCs (ratio of PM6:PSEHTT is 0.85:0.15)] are shown in Figure 2a and Figure 2b, respectively. The four photovoltaic parameters (PCE, *V*_OC_, *J*_SC,_ and FF) for all OSCs are listed in Table 1. The PM6:L8-BO-based binary OSCs have a PCE of 15.83%, a *V*_OC_ of 0.870 V, a *J*_SC_ of 24.39 mA cm^−2^, and an *FF* of 74.6%. When the ratio of PM6:PSEHTT:L8-BO is 0.9:0.1:1.4, *J*_SC_ is increased significantly (from 24.39 to 25.66 mA cm^−2^), *V*_OC_ is increased slightly (from 0.87 to 0.875 V), and FF is decreased slightly (from 74.6% to 74.2%). However, when excess PSEHTT was introduced into the PM6:L8-BO-based binary film, all photovoltaic parameters decreased simultaneously, with the high PSEHTT ternary OSC (PM6:PSEHTT:L8-BO ratio of 0.85:0.15:1.4) showing the lowest performances (the PCE of 14.37% with a *V*_OC_ of 0.870 V, a *J*_SC_ of 23.10 mA cm^−2^, and an FF of 71.5%). The *J*_SC_ values calculated from the EQE curves are 23.62 mA cm^−2^, 24.76 mA cm^−2^, and 22.25 mA cm^−2^, corresponding to PM6:L8-BO binary OSC, optimized ternary OSC, and high PSEHTT ternary OSC, respectively. The average error between the measured and calculated *J*_SC_ values is less than 4%.

The progress of ternary OSCs based on second donors used as the third component materials is summarized in Table 2. In this manuscript, while adding optimized PSEHTT into PM6:L8-BO binary films, the PCE of optimized ternary OSC is enhanced by about 5.28% compared to PM6:L8-BO binary OSC. This improvement is due to the simultaneous increase in *J*_SC_ and *V*_OC_. Both the PM6:L8-BO binary OSC and the optimized ternary OSC show similar long wavelength photocurrent response peaks, which are attributed to the stable surface and bulk morphology of the acceptor molecules. The medium wavelength photocurrent response peak of the optimized ternary OSCs is enhanced and blue shift compared to the PM6:L8-BO binary OSCs, which could be attributed to the efficient medium wavelength photon collection and blue shift absorption peaks of PSEHTT. However, the overall EQE values of the high PSEHTT ternary OSCs were reduced compared to the optimized ternary OSCs, which could be attributed to the excess PSEHTT replacing PM6, reducing exciton dissociation and charge transport.

**Figure 3:**
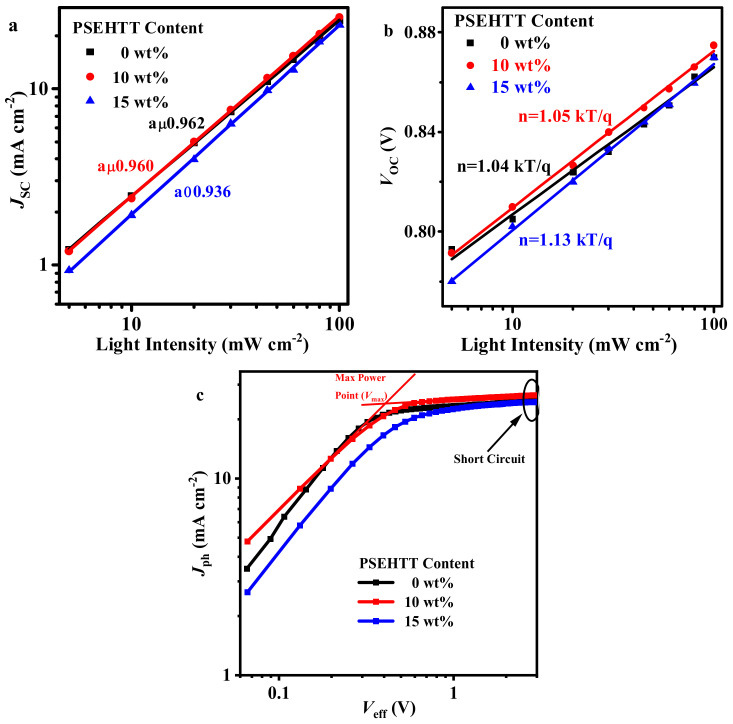
(**a**,**b**) the *J*_SC_ and *V*_OC_ values of three typical OSCs, respectively; (**c**) the *J*_ph_-*V*_eff_ curve of three typical OSCs.

To investigate the influence of the binary or ternary photoactive layer on the charge recombination mechanism, the *J*-*V* curve of three typical OSCs under different light intensities (*P*_light_) was measured (as shown in Figure S1), and the *J*_SC_ and *V*_OC_ values are shown in Figure 3a,b. The relationship between *J*_SC_ and *P*_light_ and between *V*_OC_ and *P*_light_ is given by the formula [32,33]:

$$\begin{array}{l} J_{\mathrm{SC}}\propto P_{\mathrm{light}}{}^{\alpha }\\ V_{\mathrm{OC}}\propto n\frac{kT}{q}\ln(P_{\mathrm{light}}) \end{array}$$

The bimolecular recombination under the short circuit condition and trap-assisted recombination under the open circuit condition in the photoactive layers can be evaluated from the exponential factor (α) and ideality factor (*n*) values, respectively; the bimolecular recombination and trap-assisted recombination can be considered negligible if α and *n* are very close to 1. As shown in Figure 3b, the α and *n* value deviation of optimized ternary OSCs (0.960 and 1.05, respectively) is slightly larger than PM6:L8-BO binary OSCs (0.962 and 1.04, respectively), while the α and *n* value deviation of high PSEHTT ternary OSCs (0.936 and 1.13, respectively) is significantly large than the above two OSCs. This result indicated that the low PSEHTT content in the hybrid donor produced more ideal charge recombination. While an excess amount of PSEHTT was used as the donor, it indicates that the bimolecular recombination and trap-assisted recombination in high PSEHTT ternary OSCs was very serious. Therefore, the high PSEHTT ternary OSCs showed the low photovoltaic performance [34]. The deviation of α and *n* values of optimized ternary OSCs are slightly larger than those of the PM6:L8-BO binary OSCs due to the charge recombination of optimized ternary OSCs is slightly larger than PM6:L8-BO binary OSCs. However, according to Table 2, the *V*_OC_ and *J*_SC_ of optimized ternary OSCs are both higher than those of the PM6:L8-BO binary OSCs. We believe that the *V*_OC_ and *J*_SC_ are determined using a variety of factors such as energy bandgap, light absorption, charge carrier mobility, exciton dissociation, surface and bulk morphology, and so on. Charge recombination is only one of the multiple factors affecting *V*_OC_ and *J*_SC_. We will further investigate the effect of PSEHTT as a third component on light absorption, charge carrier mobility, exciton dissociation, and surface and bulk morphology. The changes in *V*_OC_ and *J*_SC_ of the three components will be analyzed.

The photogenerated current versus the effective voltage (*J*_ph_-*V*_eff_) curves were employed to study the exciton dissociation and charge extraction processes, as shown in Figure 3c. All the parameters are summarized in Table 3. *J*_ph_ is defined as *J*_L_-*J*_D_, where *J*_L_ is the current density value under standard illumination, and *J*_D_ is the current density value without standard illumination. *V*_eff_ is defined as *V*_0_-*V*_a_, where *V*_0_ is the voltage when *J*_ph_ is 0, and *V*_a_ is the applied voltage. *P*(*E*,*T*) represents the exciton dissociation efficiency (*η*_diss_) and charge collection efficiency (*η*_coll_) of the OSCs and is given using the following equation [35]:

$$\begin{array}{l} J_{\mathrm{ph}}=J_{L}-J_{D}\\ V_{\mathrm{eff}}=V_{0}-V_{a}\\ J_{\mathrm{sat}}=qLG_{\max},\\ P(E,T)=J_{\mathrm{ph}}/J_{\mathrm{sat}} \end{array}$$

where *J*_sat_ is the photocurrent at the saturated state (when *V*_eff_ is 3 V) and *G*_max_ is the maximum exciton generation rate; all the photogenerated excitons can be dissociated, and charges can be transported and collected under the saturated state. The *P*(*E*,*T*)^a^ and *P*(*E*,*T*)^b^ are defined as *J*_ph_^a^/*J*_sat_ and *J*_ph_^b^/*J*_sat_, which represent the exciton dissociation efficiency (*η*_diss_) and charge collection efficiency (*η*_coll_), respectively [36]. The optimized ternary OSCs show a slight decrease in the *η*_diss_ and *η*_coll_ values (96.8% and 89.5%, respectively) compared to the PM6:L8-BO binary OSCs (97.0% and 89.7%, respectively), while the high PSEHTT ternary OSCs showed lowest *η*_diss_ and *η*_coll_ values (94.4% and 86.4%, respectively). This suggests that PM6:L8-BO binary OSCs and optimized ternary OSCs have similar exciton dissociation and charge transport capability, while high PSEHTT ternary OSCs have low exciton dissociation and charge transport efficiencies [37,38]. According to the above equation, the highest *J*_sat_ of optimized ternary OSCs is attributed to the efficient exciton generation rate and light absorption of optimized ternary film compared to the PM6:L8-BO binary OSCs [39,40]. The efficient photon collection in the photoactive layer is an important factor for the enhancement of photocurrent. Although the charge recombination of optimized ternary OSCs is slightly larger than that of PM6:L8-BO binary OSCs, the optimized ternary films are more efficient than the PM6:L8-BO binary films in terms of light absorption and exciton generation, which is conducive to enhancement of the *J*_SC_ values. Therefore, the *J*_SC_ values of optimized ternary OSCs are significantly larger than those of PM6:L8-BO binary OSCs.

**Figure 4:**
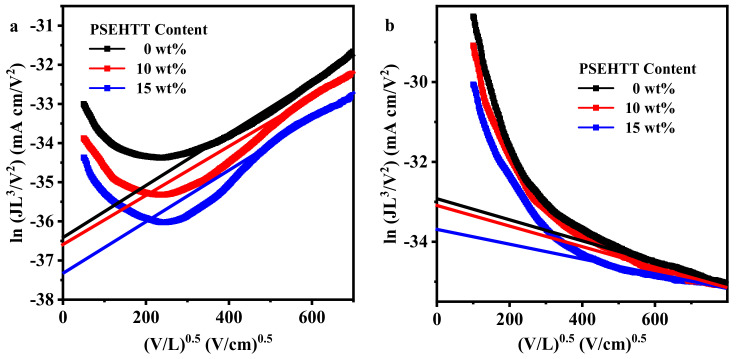
The SCLC curve of electron-only and hole-only devices corresponding to (**a**,**b**).

The space charge limited current (SCLC) method was employed to measure the hole and electron mobilities of binary and ternary SCLC devices (as shown in Figure 4 and Table 4). The electron-only and hole-only SCLC devices exhibited configurations of ITO/ZnO/photoactive layer/Ca/Al and ITO/PEDOT:PSS/photoactive layer/Au, respectively. The charge carrier mobilities were calculated using the following equation [41,42]:

$$\begin{array}{l} J=\frac{9}{8}\varepsilon_{r}\varepsilon_{0}\mu \frac{V^{2}}{d^{3}}\\ \mu =\mu_{0}\exp[0.89\gamma \sqrt{\frac{V}{L}}] \end{array}$$

where *J* is the current density, μ is the charge carrier mobility, $\varepsilon_{0}$ (8.85 × 10^−14^ F/cm) and $\varepsilon_{r}$ are the permittivity of free space and relative permittivity of the material ($\varepsilon_{r}$ was assumed to be 3), respectively, and *V* is the SCLC effective voltage. The parameter $\mu_{0}$ is the charge mobility under zero electric field, and γ is a constant. The Mott-Gurney equation can then be given as [43]:

$$\begin{array}{l} J=\frac{9}{8}\varepsilon_{r}\varepsilon_{0}\mu_{0}\frac{V^{2}}{L^{3}}\exp[0.89\gamma \sqrt{\frac{V}{L}}]\\ \ln(\frac{JL^{3}}{V^{2}})=0.89\gamma \sqrt{\frac{V}{L}}+\ln(\frac{9}{8}\varepsilon_{r}\varepsilon_{0}\mu_{0}) \end{array}$$

When a small amount of PSEHTT is doped into PM6:L8-BO binary films, the electron mobility (*µ*_e_, from 3.87 to 3.82 × 10^−4^ cm^2^ V^−1^ s^−1^) and hole mobility (*µ*_h_, from 4.45 to 4.41 × 10^−4^ cm^2^ V^−1^ s^−1^) decrease only slightly, whereas the *µ*_e_ and *µ*_h_ value of high PSEHTT ternary SCLC devices decrease significantly to 3.34 and 3.94 × 10^−4^ cm^2^ V^−1^ s^−1^.

**Figure 5:**
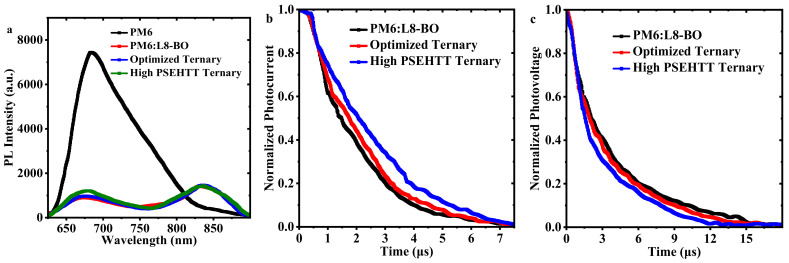
(**a**) PL spectra of the PM6 neat films, PM6:L8-BO binary films, and two ternary films (optimized ternary film and high PSEHTT ternary film); (**b**,**c**) TPC curve and TPV curve of PM6:L8-BO binary film, optimized ternary film and high PSEHTT ternary film.

The photoluminescence (PL), transient photocurrent (TPC), and transient photovoltage (TPV) were employed to investigate the effect of PSEHTT as a third component material on exciton dissociation and charge carrier recombination in both binary and ternary films. The PM6 neat film showed a stronger PL spectrum, while the PM6 PL peak was quenched strongly, and the L8-BO PL peak appeared slightly for the PM6:L8-BO binary film. Both the PM6:L8-BO binary film and the optimized ternary film show similar PM6 residual PL peaks and the L8-BO PL peaks, which suggests that exciton dissociation is stable in the presence of a small amount of PSEHTT as the third component material. However, for high PSEHTT ternary films, the PM6 residual PL peaks are significantly enhanced compared to the optimized ternary films, suggesting that excess PSEHTT disrupts exciton dissociation. To further understand the charge extraction/recombination kinetics of the binary and ternary films, we measured TPC and TPV to calculate the charge extraction time and carrier lifetime, respectively. As shown in Figure 5b, the charge extraction times of PM6:L8-BO binary and optimized ternary films are 0.261 μs and 0.265 μs, respectively, while the charge extraction time of the high PSEHTT ternary film is even longer at 0.375 μs. The similar charge extraction times for PM6:L8-BO binary films and optimized ternary films indicate similar charge carrier transport capabilities. The high PSEHTT ternary film has a longer charge extraction time, indicating a lower charge carrier mobility. As shown in Figure 5c, the carrier lifetimes of PM6:L8-BO binary and optimized ternary films are 4.21 μs and 4.05 μs, respectively, which are longer than that of the high PSEHTT ternary film (3.13 μs). Compared to the PM6:L8-BO ternary film, the weaker reduced charge carrier lifetime for the optimized ternary film suggests that the effect of charge carrier recombination is weaker changed in the presence of a small amount of PSEHTT as the third component material [44]. The significant increase in charge extraction times and obviously enhanced charge carrier lifetime for the high PSEHTT ternary film indicate that the over amount of PSEHTT has damaged the charge carrier kinetics.

It is commonly recognized that the *V*_OC_ of binary OSCs mainly depends on the energy level difference between the LUMO of the acceptor and HOMO of the donor (*E*_g_) as well as the energy loss (*E*_loss_) according to the empirical relation shown below [11]:

$$V_{\mathrm{OC}}=\frac{E_{g}^{}}{e}-\frac{E_{\mathrm{loss}}}{e}$$

However, after incorporating the third component materials into binary films, the HOMO energy levels of the hybrid donor will be shifted. In addition, the *E*_loss_ value will be inevitable in photoelectric conversion processes. PSEHTT showed deeper HOMO energy levels than PM6 (the HOMO levels are −5.45 and −5.25 eV, respectively), which is beneficial to form the cascade HOMO energy levels. The energy levels of the hybrid donor are calculated according to the empirical relation shown below [45]:

$$E_{\mathrm{Blend}}=\frac{f_{\mathrm{PM}6}\cdot N_{e}{}_{\mathrm{PM}6}\cdot E_{\mathrm{PM}6}+f_{\mathrm{PSEHTT}}\cdot N_{e}{}_{\mathrm{PSEHTT}}\cdot E_{\mathrm{PSEHTT}}}{f_{\mathrm{PM}6}\cdot N_{e}{}_{\mathrm{PM}6}+f_{\mathrm{PSEHTT}}\cdot N_{e}{}_{\mathrm{PSEHTT}}}$$

where *f* is the weight ratio, *N*_e_ is the quasi-frontier orbital density, and *E* is the energy level value. The energy levels of the relevant materials are calculated from other papers [46,47]. The HOMO energy levels of the hybrid donor are calculated according to the abovementioned empirical relation. *N*_e_ can be calculated according to the following formula [48]:

$$N_{e}=nl$$

where *n* and *l* are the molecular number of the unit mass and the number of quasi-degenerate HOMOs per molecule (<0.1 eV) of the donor, respectively, the molecular weight, *n*, *l*, and *N*_e_ values of neat PSEHTT and PM6 are list in Table S1. As shown in Table S2, along with the increase in PSEHTT content in the hybrid donor, a downshift of HOMO energy levels and a broadening of *E*_g_ value is also observed, which is beneficial to the enhancement of *V*_OC_ values of the ternary OSCs. However, the *V*_OC_ values of the ternary OSCs decrease when an excess amount of PSEHTT is added to the PM6:L8-BO binary films, which could be attributed to the variation of phase separation and surface morphology. Although the charge recombination of optimized ternary OSCs is slightly large than that of PM6:L8-BO binary OSCs, the HOMO energy levels of the blend donor are obviously downshifting, and the energy bandgap is obviously broadening, which is conducive to the enhancement of the *V*_OC_ values of optimized ternary OSCs. Therefore, the *V*_OC_ values of optimized ternary OSCs are slightly larger than those of PM6:L8-BO binary OSCs.

The phase separation and surface morphology of the binary and ternary films were characterized using transmission electron microscope (TEM) and atomic force microscope (AFM), respectively. The three typical films (PM6:L8-BO binary film, optimized ternary film, and high PSEHTT ternary film) showed different molecular aggregations. As shown in Figure S2a–c, the light and dark domains represent the donor and acceptor domains, respectively. The optimized ternary film shows only slight changes in the light and dark domains, with slightly enhanced molecular aggregation and homogeneous morphology compared to the PM6:L8-BO binary film, whereas the high PSEHTT ternary films show significant molecular aggregation and inhomogeneous morphology, which supports the low FF values. As shown in Figure S2d–f, the root-mean-square (RMS) roughness value of the optimized ternary film is about 0.98 nm, which is slightly higher than that of the PM6:L8-BO binary film (0.96 nm) and significantly lower than that of the high PSEHTT ternary film (1.15 nm). The smooth surface of the optimized ternary film further confirms its homogeneous morphology, which facilitates charge transport and collection.

In order to further investigate the effect of PSEHTT content in the hybrid donor on the molecular arrangement of the ternary films, X-ray diffraction (XRD) was performed. The angles at which the peak intensities occur are related to the interplanar distances of the atomic structure of the photoactive layer and the crystallinity of the photoactive layer; these angles are related to Bragg’s law [49]:

λ=2dsinθ

where *λ* is the wavelength of the X-ray radiation (0.154 nm), *θ* is the peak position half-angle, and *d* is the interplanar distance. The XRD spectra of neat PM6, PSEHTT, and L8-BO films are shown in Figure S3a. Both PM6 and PSEHTT show strong (100) and slight (010) diffraction peaks in the XRD spectra. The L8-BO film shows the opposite XRD profile (weak (100) and strong (010) diffraction peaks) compared to the PM6 and PSEHTT films. Figure S3b shows the XRD curves for three typical hybrid films (PM6:L8-BO binary film, optimized ternary film, and high PSEHTT ternary film). The (100) diffraction peak intensities of the optimized ternary film and the high PSEHTT ternary film are slightly stronger than that of the PM6:L8-BO binary film, which is attributed to the fact that the (100) diffraction peak intensity of PSEHTT is stronger than that of PM6 [50,51].

## 3. Experimental Details

PM6, PSEHTT, and L8-BO were purchased from Solarmer Materials Inc. (El Monte, CA, USA) Chloroform (CF) and isopropanol were purchased from Sigma-Aldrich Co. (St. Louis, MO, USA) PEDOT:PSS, clevios PVP Al 4083 was purchased from H.C. Starck Co., Ltd. (Rayong, Thailand) Al, and Ag were purchased from Alfa Aesar Co. (Heysham, UK). The ratio of donor:acceptor was kept constant (1:1.4) in all binary and ternary OSCs, and the PSEHTT content in donor blends was adjusted from 0 wt% to 15 wt%. The blend of PM6, PSEHTT, and L8-BO was dissolved in CF as a function of the ratio of PM6:PSEHTT (the blend materials concentration is 14 mg mL^−1^ in total) with the solvent additive of 1-chloronaphthalene (CN) (0.5%, *v*/*v*).

Indium–tin-oxide (ITO) glasses were ultrasonicated at 30 °C in isopropyl alcohol, acetone, and deionized water for 30 min. The PEDOT:PSS solution was spin-coated onto the ITO glass and baked at 150 °C for 20 min in the air (the thicknesses of the PEDOT:PSS thin films are 20 nm). The photoactive blend solution was spin-coated on the PEDOT:PSS layer in an N_2_-filled glove box, and the thermal annealing treatment was carried out (with a nominal thickness of ~100 nm). Then, the solution of PFN-Br was dissolved in methanol with a concentration of 0.5 mg/mL and spin-coated over the active layers at 3000 rpm for 35 s. Finally, the Al films (120 nm) were sequentially deposited on top of the photoactive layer by thermal evaporation, and the photoactive area was 4 mm^2^ (2 × 2 mm^2^).

The current density versus voltage (*J*-*V*) characteristics were measured in a glove box with a computer-controlled Keithley 236 Source Measure Unit under illumination at 100 mW cm^−2^ using an AM 1.5 G solar simulator. Average photovoltaic parameter values were obtained from ten devices fabricated in parallel. The *EQE* spectrum was measured with a Stanford Research Systems model SR830 digital signal processor (DSP) lock-in amplifier coupled to a WDG3 monochromator and a 500 W xenon lamp.

## 4. Conclusions

In summary, ternary OSCs were fabricated using neat L8-BO as the electron acceptor and a blend of PM6 and PSEHTT as the donor. The PM6:L8-BO-based binary OSCs showed a PCE of 15.83%, a *V*_OC_ of 0.870 V, a *J*_SC_ of 24.39 mA cm^−2^, and an FF of 74.6%. The highest photovoltaic performance of ternary OSCs (PCE of 16.66%, with *V*_OC_ of 0.875 V, *J*_SC_ of 25.66 mA cm^−2^, and FF of 74.2%) was achieved when the ratio of PM6:PSEHTT:L8-BO was 0.9:0.1:1.4. The slight enhancement of *V*_OC_ was due to the deeper HOMO level of PSEHTT than that of PM6, which broadens the optical bandgap. We conclude that a small amount of PSEHTT replaces PM6 to achieve efficient light absorption and broaden the optical bandgap while maintaining the surface and bulk morphology of the ternary film. However, an excessive amount of PSEHTT instead of PM6 disrupts the surface and bulk morphology of the ternary films, which is detrimental to exciton dissociation charge carrier transport and enhances charge carrier recombination. The results suggest that the ternary strategy is an effective method to further improve the performance of OSCs.

## Figures and Tables

**Table 1:** Photovoltaic parameters of ternary OSCs as a function of the ratio of PM6:PSEHTT.

PM6: PSEHTT	*V_OC_* (V)	*J_SC_* (mA cm^−2^)	*FF* (%)	*PCE* (%)
100:0	0.870	24.39 ± 0.13	74.6 ± 1.18	15.83 ± 0.12
95:5	0.872	25.09 ± 0.14	74.4 ± 1.22	16.28 ± 0.13
92.5:7.5	0.874	25.28 ± 0.14	74.3 ± 1.27	16.42 ± 0.12
90:10	0.875	25.66 ± 0.13	74.2 ± 1.21	16.66 ± 0.13
87.5:12.5	0.877	25.68 ± 0.14	73.3 ± 1.18	16.51 ± 0.15
85:15	0.870	23.10 ± 0.13	71.5 ± 1.17	14.37 ± 0.14

**Table 2:** The PCE of ternary PSCs based on second donors as third component materials.

Binary Hybrid (D:A)	PCE (%)	Third Component (%)	PCE (%)	Increased Ratio (%)	References
PM6:BTP-eC9	17.34	BPR-SCl (20%)	18.02	3.92%	[24]
PM6:Y6	16.47	TTBT-R (10%)	18.07	9.71%	[25]
PM6:L8-BO	17.55	BTC (15%)	18.24	3.93%	[26]
PM6:C9	17.38	PM6-Si30 (15%)	18.27	5.12%	[27]
D18-Cl:Y6	17.35	G19 (10%)	18.53	6.80%	[28]
PM6:BTP-eC9	17.30	PB2F (10%)	18.60	7.51%	[29]
PM6:L8-BO	17.63	BTID-2F (10%)	18.52	5.05%	[30]
PM6:L8-BO	18.20	D18 (20%)	19.60	7.69%	[31]

**Table 3:** *J*_ph_, *J*_sat_, and P (E, T) under short-circuit and maximum power-point conditions of the three OSCs (^a^ short-circuit condition, ^b^ maximum power-point condition).

PM6: PSEHTT	*J*_ph_^a^ (mA cm^−2^)	*J*_ph_^b^ (mA cm^−2^)	*J*_sat_ (mA cm^−2^)	*G*_max_ (m^−3^s^−1^)	P (E, T) ^a^ (%)	P (E, T) ^b^ (%)
100:0	24.39	22.55	25.14	1.571 × 10^28^	97.0	89.7
90:10	25.66	23.72	26.51	1.657 × 10^28^	96.8	89.5
85:15	23.10	21.14	24.47	1.530 × 10^28^	94.4	86.4

**Table 4:** Electron mobility (μ_e_), and hole mobility (μ_h_) of three typical SCLC devices.

PM6: PSEHTT	μ_e_ (cm^2^ V^−1^ s^−1^)	μ_h_ (cm^2^ V^−1^ s^−1^)
100:0	3.87 × 10^−4^	4.45 × 10^−4^
90:10	3.82 × 10^−4^	4.41 × 10^−4^
85:15	3.34 × 10^−4^	3.94 × 10^−4^

## Data Availability

If necessary, the corresponding author can provide the research data, please contact the corresponding author.

## Supplementary Materials

The following supporting information can be downloaded at: https://www.mdpi.com/article/10.3390/molecules28196832/s1, Figure S1. J-V characteristics under various light intensities ranging. Table S1. The molecular parameter of PM6 and PSEHTT. Table S2. The energy level and optical bandgap parameter of ternary photoactive layer. Figure S2. TEM and AFM images of photoactive layer. Figure S3. The XRD profiles of neat films and blended films.

## Sample Availability

Samples of the compounds are available from the authors.
