# Supplementary material for: Ternary Organic Solar Cells by Small Amount of Efficient Light Absorption Polymer PSEHTT as Third Component Materials
Source: Molecules. 2023 Sep 27;28(19):6832. doi: 10.3390/molecules28196832 (PMC10574318; doi:10.3390/molecules28196832)
Supplement: Supplementary file 1 [file molecules-28-06832-s001.zip › molecules-2629885-supplementary.pdf]

Supporting Information

Ternary Organic Solar Cells by Small Amount of Efficient Light Absorption Polymer PSEHTT as Third Component Materials

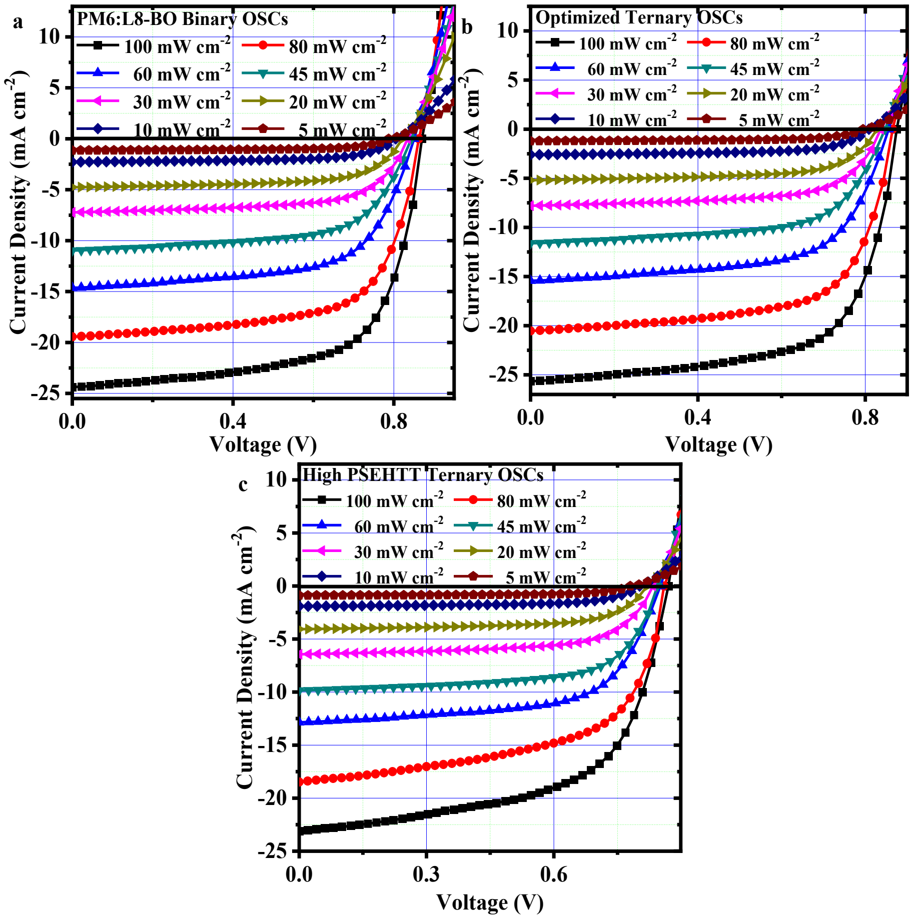

Figure S1. *J-V* characteristics under various light intensities ranging from 100 mW cm^{-2} to 5 mW cm^{-2} for the PM6:L8-BO binary OSCs, optimized ternary OSC and high PSEHTT ternary OSCs corresponding to Figure S1(a), S1(b) and S1(c), respectively.

Table S1. Summary of the molecular weight, n, l, and N_e values of PM6 and PSEHTT.

Donor	Molecular Weight (g mol^{-1})	n (mol g^{-1})	l	N_e (mol g^{-1})	E_{HOMO} (eV)	E_{LUMO} (eV)
PSEHTT	975	6.17×10^{20}	1	6.17×10^{20}	-5.45	-3.30
PM6	1206	4.99×10^{20}	1	4.99×10^{20}	-5.25	-3.06

Table S2. The HOMO energy level and optical bandgap of ternary photoactive layer as a function of the PM6:PSEHTT ratio.

PM6: PSEHTT	E_{HOMO} (eV)	E_{g} (eV)
100:0	-5.250	1.150
95:5	-5.258	1.158
92.5:7.5	-5.262	1.162
90:10	-5.266	1.665
87.5:12.5	-5.270	1.171
85:15	-5.275	1.175

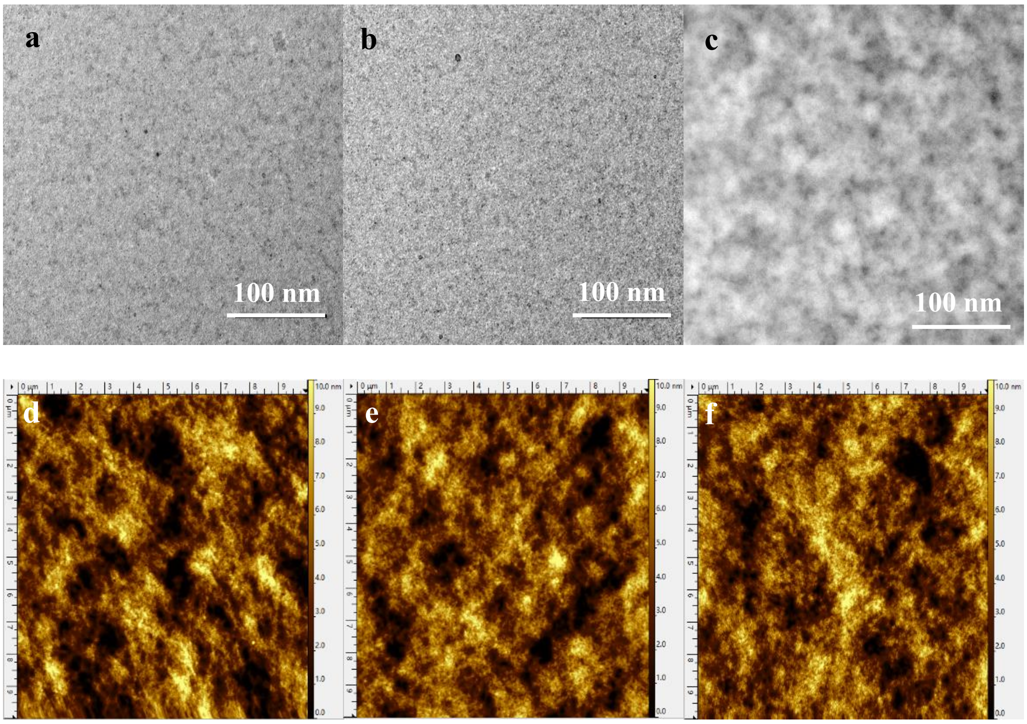

Figure S2. TEM (a, b and c) and AFM (d, e and f) images of the PM6:L8-BO binary film, optimized ternary film and high PSEHTT ternary film, respectively.

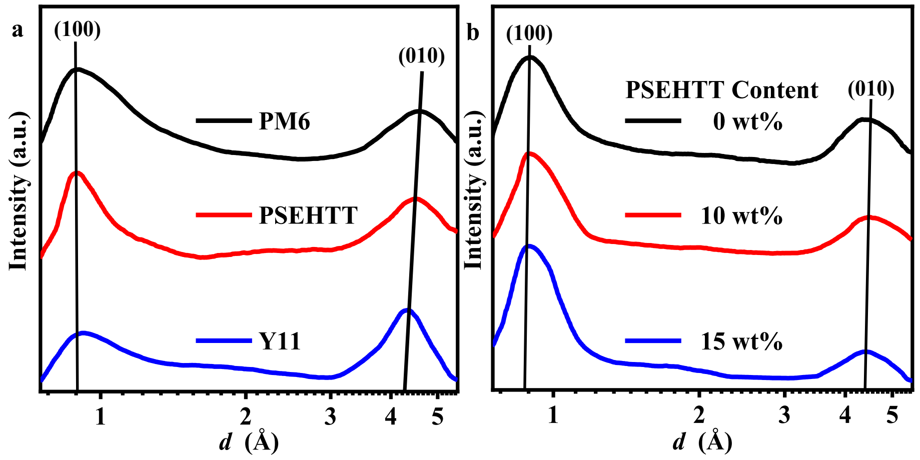

Figure S3. (a) The XRD profiles of neat PM6, PSEHTT and L8-BO films. (b) The XRD profiles of three typical blended films (PM6:L8-BO binary film, optimized ternary film and high PSEHTT ternary film).
